# Effect of Janus Nanosheets in Polypropylene on Rheological Properties and Autoclave Foam Performance

**DOI:** 10.3390/polym15163433

**Published:** 2023-08-17

**Authors:** Yaohui Xu, Peng Guo, Hengyuan Zhang, Minqiao Ren, Mingfu Lyu

**Affiliations:** SINOPEC Beijing Research Institute of Chemical Industry, Beijing 100013, China; xuyh.bjhy@sinopec.com (Y.X.); guopeng.bjhy@sinopec.com (P.G.); zhanghy.bjhy@sinopec.com (H.Z.); renmq.bjhy@sinopec.com (M.R.)

**Keywords:** polypropylene, foam autoclave, rheological properties, Janus nanosheet, cell structure

## Abstract

Our experiment revealed that the addition of Janus nanosheets to polypropylene (PP) has a significant impact on the viscoelasticity of the composite system. Specifically, when 0.10 wt% of Janus nanosheets were added, the complex viscosity of the composite system increased. However, when we added less than 0.05 wt% of Janus nanosheets, there was a reduction in complex viscosity, which is known as the non-Einstein phenomenon. The Cole–Cole plot showed that the nanosheet network structure did not have a significant effect on the viscosity of the composite system. Additionally, we used carbon dioxide as a foaming agent to autoclave foaming using modified PP from Janus nanosheets, and the results demonstrated that increasing the number of Janus nanosheets decreased the apparent density and strengthened the cell structure of foaming beads, resulting in improved closed porosity.

## 1. Introduction

The polymer community has paid close attention to polymeric foams because they satisfy the criteria for sustainable development when taking into account energy savings, emission reduction, and the circular economy [1,2]. Expanded polypropylene (EPP) beads are utilized in a variety of applications, including new energy vehicles, 5G communications, drones, logistics, heat preservation, precision equipment, and electronics turnover packaging, because of their special benefits in terms of thermal performance, low weight, and environmental friendliness [3,4,5,6]. The autoclave batch-foaming method for creating EPP results in a material with a greater closed cell ratio and superior mechanical characteristics. Particularly, the blowing agent is the secure and ecologically favorable carbon dioxide [7,8,9,10].

The behavior of the melt and, eventually, the qualities of the foam are influenced by temperature, pressure, and the blowing agent during the EPP process [11,12]. After the melt exits the autoclave’s bottom unload valve, the viscoelastic qualities come into play because, during the foaming stage, the melt is prone to distortion due to cell development. The production of low-density foams is significantly complicated by the rheology of PP. PP typically has a lower viscosity in its completely melted condition than amorphous polymers like polystyrene [13,14,15].

Optimizing the viscoelastic properties of the melt is a crucial factor in achieving optimal cell development and preventing the rupture of developing cell walls. To achieve the desired foam shape, it is imperative to consider the viscoelastic behavior of the melt during processing flow field deformation. This is because achieving small average cells, a narrow cell size distribution, and low density is key to obtaining the best possible outcome [16,17,18]. The viscoelastic characteristics of standard-grade PP are generally recognized as quite unfavorable. Even though EPP does not need the same high melt strength and drawability as PP foam that is extruded, increasing its complex viscosity should still have a favorable impact on the foaming characteristics and cell structure of the material.

The foam morphology of linear PP was unsatisfactory due to excessive cell coalescence, causing most cells to open and create linkages within the foam. However, the ultimate cell density for linear PP was higher than other techniques, such as extrusion foaming, when the EPP bead foams were quenched using water to reduce bubble coalescence. The key to creating excellent linear PP foams is to suppress the cell coalescence caused by the weak melt viscoelastic characteristics of linear PP.

Numerous investigations have explored ways to make up for the poor melt viscoelastic characteristics of PP [19,20]. For example, commercial branching PP was created to create high-quality PP foams [21,22]. PP with branching exhibits excellent melt strength and effectively minimizes cell coalescence, making it the preferred choice for manufacturing extruded PP foam products. Nevertheless, the presence of extended-branched molecular chains may cause molecular weight to rise and mobility to decrease [23,24]. The MFI of branching PP is often less than 3 g/10 min (230 °C with 2.16 kg), which is insufficient to fulfill the requirements of the EPP process, and its cost is significantly greater than that of linear PP [25]. Therefore, the use of branching PP is frequently avoided.

Studies have found that adding inorganic particles [26,27,28], particularly nanoparticles, to PP foams can greatly aid in cell nucleation. These particles have been successfully used in the EPP foaming process as efficient nucleating agents, which help to reduce foam cell size and increase cell density. It is important to ensure that the particles are well dispersed throughout the polymer melt. Previously, micrometer-sized fillers such as talc, clay, and SiO_2_ sheets were used for dispersion. However, the inorganic microparticles needed for EPP differ from traditional microfillers in that they not only aid in cell nucleation but also play a significant role in promoting viscoelastic properties of the polymer [29,30].

Besides the viscoelasticity of the raw material, the apparent density of EPP beads and the structure of the cell are also influenced by the nucleation and growth ability of the bubble cell during the pressure relief process, as the foaming agent escapes from the raw material. From some prior research [31,32], we knew that adding nanoparticles in amounts of 1.0 wt% or less does not alter the melt viscosities or relaxation time of polymer chain entanglement. However, when specific nanoparticles are included in polymer nanocomposites, they can significantly affect the rheological behaviors of polymers and the resulting foam properties [33,34].

The use of Janus particles [35,36], which have two different compositions, on a single surface has become popular in both academic and industrial fields due to their diverse and promising performances. The anisotropic shape of these particles can provide additional gains by making the polymer melt more viscoelastic. This is due to the restricted polymer chain rotation that occurs at the interface between the melt and nanosheets [37,38]. Inorganic Janus nanosheets have recently been created through multiple lithography etching onto silicon or crushing modified glass hollow spheres.

Janus nanosheets are a useful tool for addressing interfacial engineering issues at various length scales. The composition, size, and microstructure of these sheets are crucial in determining how they disperse among PP chains as viscoelastic extenders and form complex structures as foam cell wall building blocks. However, adjusting Janus nanosheets to achieve optimal results remains difficult [39].

Our study focuses on the effects of adding Janus nanosheets to PP compared to neat PP and PP/talc. We examine the preparation, complex viscosity, and batch-foaming ability of these materials, as well as the impact of batch-foaming processes on the morphology and microstructure of the EPP beads. Additionally, we compare the viscoelastic properties of PP with different types and amounts of nanosheet. We also discuss the mechanism of Janus nanosheet entanglement with PP molecular chains, which differs from other inorganic particles. Finally, we compare the apparent density and cell wall morphology structures of EPP beads supplemented with different inorganic particles, highlighting the benefits of adding Janus nanosheets in terms of lower density and improved cell structure quality, particularly when used in specific thickness and proportion.

## 2. Materials and Methods

### 2.1. Materials

In the scope of this work, two linear ethylene-propylene random copolymer grades were studied: BHY-PP was polymerized and provided by Sinopec, Beijing research institute of the chemical industry. BHY-PP has MW of 286, 901 g/mol (PDI of 5.50), and MFI of 7.1 g/10 min (230 °C with 2.16 kg). The Sinopec materials are chemically pure. Thus, additives can be excluded. 

**Figure 1:**
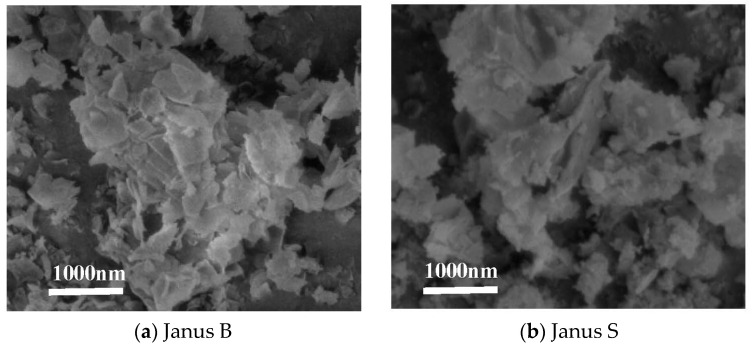
SEM images of Janus B and Janus S.

There are two Janus nanosheets with the same functional groups (one side grafting poly(methyl methacrylate), the other side grafting amidogen) produced and provided by TsingHua University, They will be referred to as Janus B with an average width of 1000–2000 nm and thickness of 100 nm and Janus S with an average width of 1000–2000 nm and thickness of 200 nm (Figure 1).

MICROTUFF AG609 (herein termed “talc”) is manufactured by Minerals Technologies Inc and produced in Barretts, Montana. Its median particle size is 1.0 μm. The physical blowing agent, CO_2_ (99.9% purity), was made available by Jinghui gas company, Beijing, China.

### 2.2. Sample Preparation

We used a twin-screw extruder (Coperion, CTE35, Stuttgart, Germany) and an underwater micropelletizer system (Nordson, Labline 100, Erkrath, Germany) to create PP micropellets that were around 1 mm in diameter [40]. Ten different batches containing selected concentrations of additives and two neat PP batches were prepared in amounts of 10 kg each (Table 1). The overview of the compounded material and pelletizing parameters are available in Table 1. Our study employed talc sheet or Janus nanosheets as a foaming agent, as well as other functional additives such as antistatic agent (Atmer122 0.50 wt%) and antioxidants (Irganox 1010 0.20 wt%), which were well mixed. The micropellets were then screened using a vibrating screen to remove any undersized or oversized micropellets.

### 2.3. Rheological Measurements

In order to assess the rheological properties, we used a rotational rheometer (Anton Paar, MCR302, Graz, Austria) with a parallel-plate geometry (25 mm diameter). We conducted small-amplitude oscillatory shear tests at 180 °C, with frequencies ranging from 0.01–100 rad/s. We applied a 1% strain, which fell within the linear viscoelastic range for all samples.

### 2.4. Autoclave Foaming Experiments and Characterization

The process of creating foam batches involved combining micropellets with stabilizers and water in an autoclave, introducing carbon dioxide, and gradually increasing the temperature until the micropellets melted. After reaching CO_2_ saturation, the micropellets were rapidly released to expand into EPP beads. The apparent density can be adjusted by regulating temperature and pressure [41,42]. The beads were washed with recycled softened water to remove stabilizers and post-annealed at 70 °C for 4 h before being stored in a tank for gas exchange. 

The apparent densities of the samples before and after foaming were measured using the water displacement method in accordance with the ASTM D792-13 [43]. 

The closed porosity of the EPP was determined using a true density meter (Quantachrome, UPYC1200e, Boynton Beach, FL, USA), based on Archimedes’ and Bohr’s laws (PV = nRT), in accordance with the ASTM D6226-15 standard [44]. 

To determine the Janus nanosheet distribution in modified PP and the cellular structure of the EPP beads, scanning electron microscopy (SEM; FEI SL-30, Waltham, MA, USA) was used to characterize the morphology. The samples were cut into two parts using a sharp blade, and the fracture surfaces were sputter-coated with platinum. The SEM micrographs were used to determine the average cell size.

## 3. Results and Discussion

Our first priority is to thoroughly analyze and characterize the samples. Following that, we present and explain the ultimate morphology of the EPP foam, utilizing the characteristic properties of the Janus nanosheets/PPs.

### 3.1. Structure of PP/Janus Nanosheets

In Figure 1, it is evident that both Janus B and Janus S are experiencing severe agglomeration. This is due to various factors, such as the interaction of different surface functional groups on both sides, causing the nanoparticles to be mutually attracted. Additionally, the distance between the nanosheets is very short, making the van der Waals force between them much stronger than their own gravity, resulting in their attraction to one another and agglomeration. Due to the large surface area and high surface energy, Janus nanosheets are in an unstable state and tend to aggregate to achieve stability. To address this issue, we have developed a processing flow field using microparticles to promote even dispersion of Janus nanosheets in polypropylene resin.

Table 1 shows that the addition of talc and Janus nanosheets, regardless of their shape or quantity, has no impact on the pelletizing parameters of neat PP and modified PP. We can now focus on how the processing flow field affects the dispersion state of Janus nanosheets. Based on our design, the process of the Janus/PP combination is a hybrid system with multiple phases and components that will undergo change. The Janus piece has two sides—one that works with PP and the other side that is repelled by it. It has a great ability to regulate the interface between the two. In a specific processing system, agglomerated flake particles can bend due to the movement of the polymer fluid, which overcomes the bending modulus Eb of the fragment, resulting in them bending and separating from each other.

**Figure 2:**
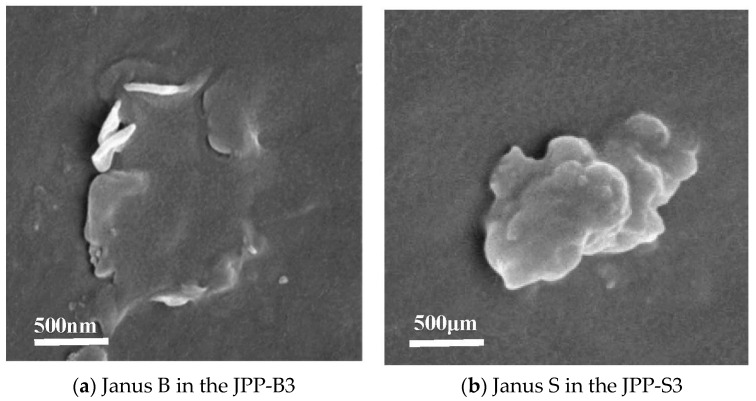
SEM images of Janus B or Janus S distributed in the modified PP.

By examining Figure 2, it is evident that the screw shear at high temperature does not damage the monolithic structure of Janus B and Janus S. In fact, their dispersion is actually enhanced in the processing flow field. Janus B enables the observation of the distribution of individual nanosheets in PP, whereas Janus S is still observed as several layers.

### 3.2. Linear Viscoelastic Properties

Linear viscoelastic properties are susceptible to the structural change in the materials. It will be demonstrated below from different rheological plots that the Janus-modified PP systems can result in new molecular chain entanglement mode with shorter or longer relaxation time, which is ascribed to the particular shape and the influence of different functional groups on both sides of Janus nanosheets.

**Figure 3:**
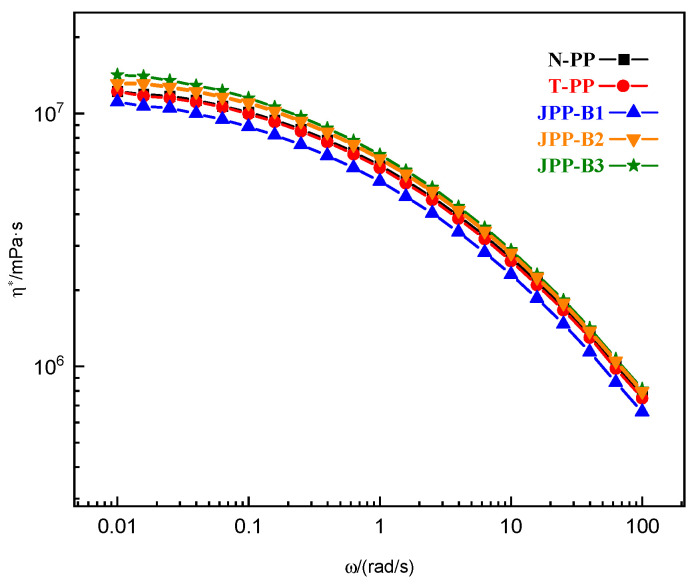
Complex viscosity vs. angle frequency for the N-PP, T-PP and Janus B modified PPs at 180 °C.

The way in which molecules are interconnected greatly affects shear rheology. Typically, even when a very small number of inorganic sheets are present, the zero-shear viscosity (η0) remains unchanged compared to pure polymers. The complex viscosity curves for N-PP, T-PP, and Janus B modified PPs are depicted in Figure 3. When only 0.30 wt% talc was used in sample T-PP, there was little effect on the complex viscosity as the amount of talc was insufficient to impact the melt’s flow and was too low to influence the infiltration network in PP. If Janus B was added in concentrations greater than 0.10 wt%, the complex viscosity curves showed higher values than those of N-PP at lower frequencies. This suggests that Janus B might have hindered the movement of PP chains, changed the relaxation mechanism, and created a percolation structure. Furthermore, the use of more Janus B resulted in a gradual increase in PP viscosity at low frequency, and the transition from a Newtonian plateau to a shear thinning regime was shifted to a lower frequency.

With the addition of 0.05 wt% of Janus sheets, a “viscosity reduction” effect with non-Einstein behavior occurred. The small number of Janus sheets prevented them from touching each other, and their size was smaller than the space in which polymer chains entangle. It is possible that the Janus sheets absorbed the molecular chains on their surface. This absorption may have led to more free space at the interface, which could have altered the structure of entanglement and reduced its density. 

Inorganic microparticles typically do not form a percolation structure in PP at such low addition rates, which would alter the composite system’s phase composition. However, Janus nanosheets have a relatively larger specific surface area and diameter-thickness ratio, so a Cole–Cole plot was used for investigation.

**Figure 4:**
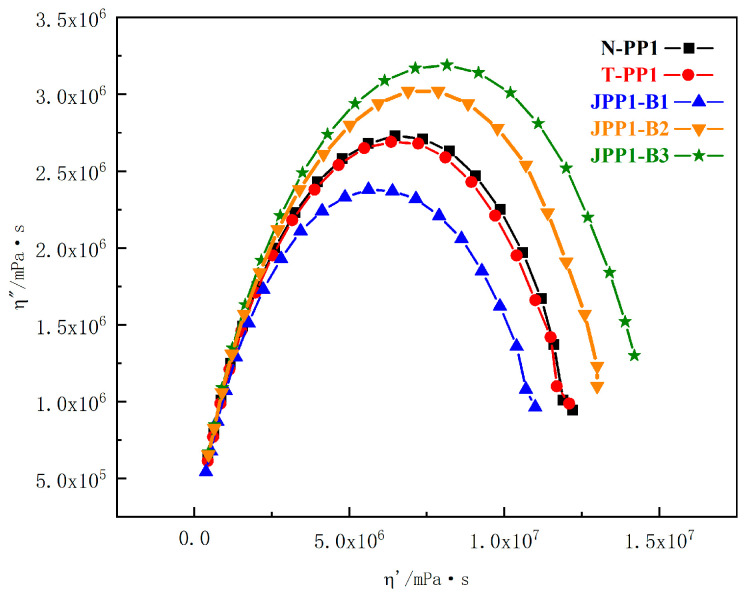
Cole–Cole plot for the N-PP, T-PP, and Janus B modified PPs at 180 °C.

The Cole–Cole plot (Figure 4) clearly demonstrates this phenomenon with minimal differences between the samples. The Cole–Cole plots for N-PP, T-PP, and Janus B modified PPs were almost semicircular, with a larger radius indicating a higher complex viscosity. The curves of N-PP and T-PP were nearly identical, lower than those of JPP-B2 and JPP-B3, and higher than JPP-B1, suggesting the presence of Janus nanosheets without longer relaxation processes at high viscosity in the modified PPs. It is important to note that the concentration of Janus B has not yet reached the point at which a continuous percolation network forms in the PP.

**Figure 5:**
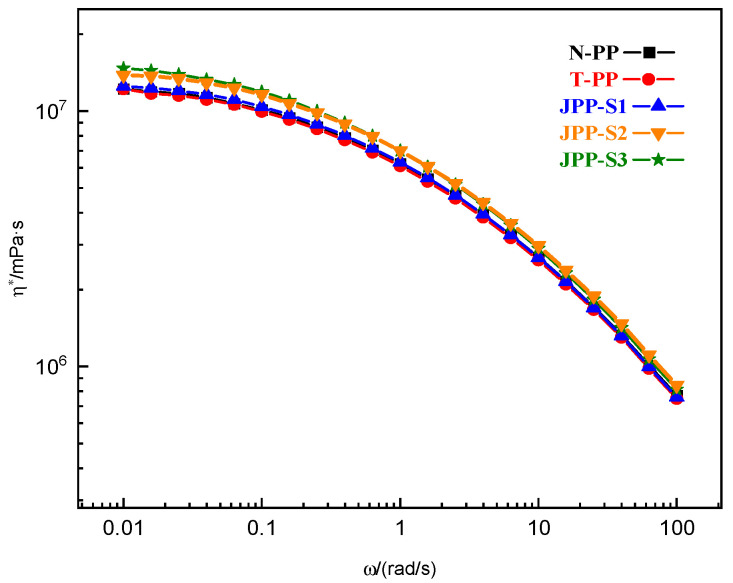
Complex viscosity vs. angle frequency for the N-PP, T-PP, and Janus S modified PPs at 180 °C.

**Figure 6:**
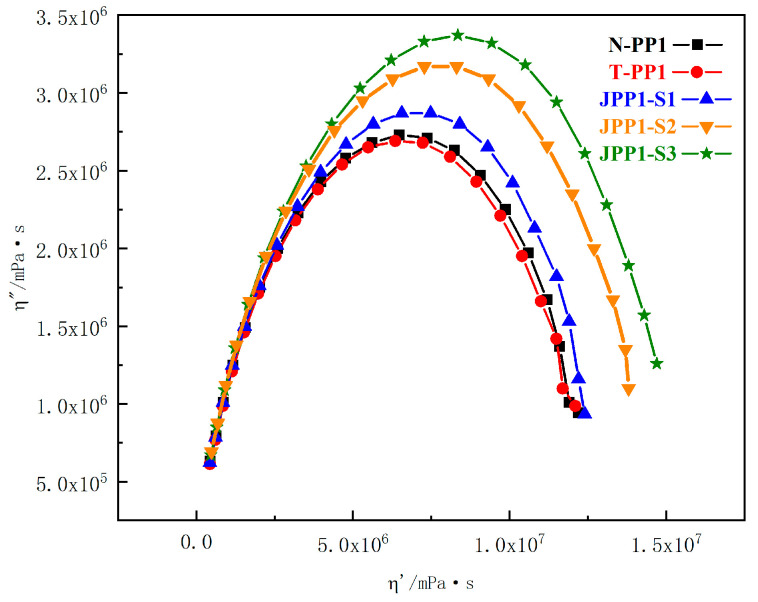
Cole–Cole plot for the N-PP, T-PP, and Janus S modified PPs at 180 °C.

Figure 5 and Figure 6 clearly demonstrate the impact of Janus S modification on the complex viscosity and Cole–Cole plot of PP. It is important to note that exceeding a Janus S content of 0.10 wt% leads to a significant increase in complex viscosity or semicircle radius. Conversely, a Janus S addition of 0.05 wt% does not alter the complex viscosity of JPP-S1. It has been shown that the thickness of the Janus nanosheets has a notable impact on the viscoelasticity of the composite system. The decrease in apparent viscosity only occurs when the Janus nanosheet thickness is below a particular threshold.

**Figure 7:**
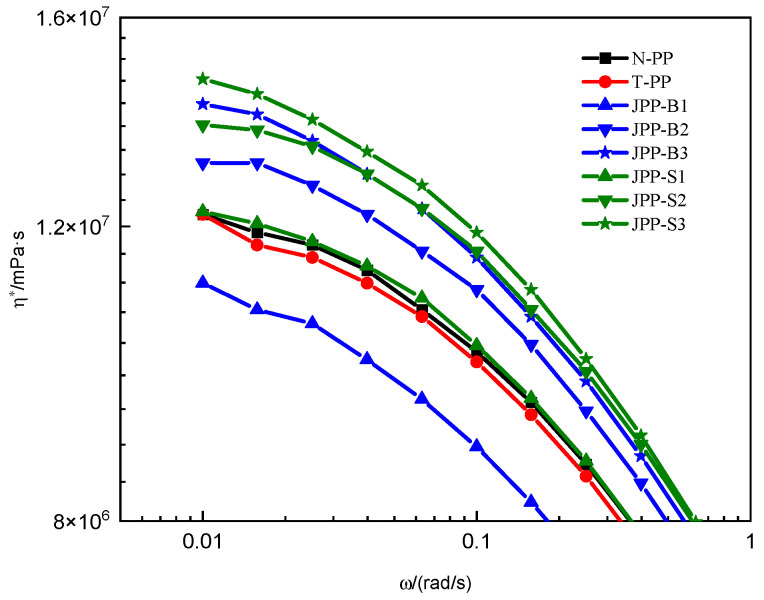
Complex viscosity vs. angle frequency for the NPP-1 serial PPs at 180 °C in 0.01–1 rad/s.

Figure 7 presents the complex viscosity vs. angle frequency curves of all samples in the lower frequency zone (0.01–1 rad/s). The findings clearly demonstrate that the complex viscosity of Janus S increases to a greater extent than that of Janus B as the number of Janus nanosheets added rises. Additionally, shear thinning occurs earlier in the case of Janus S.

**Figure 8:**
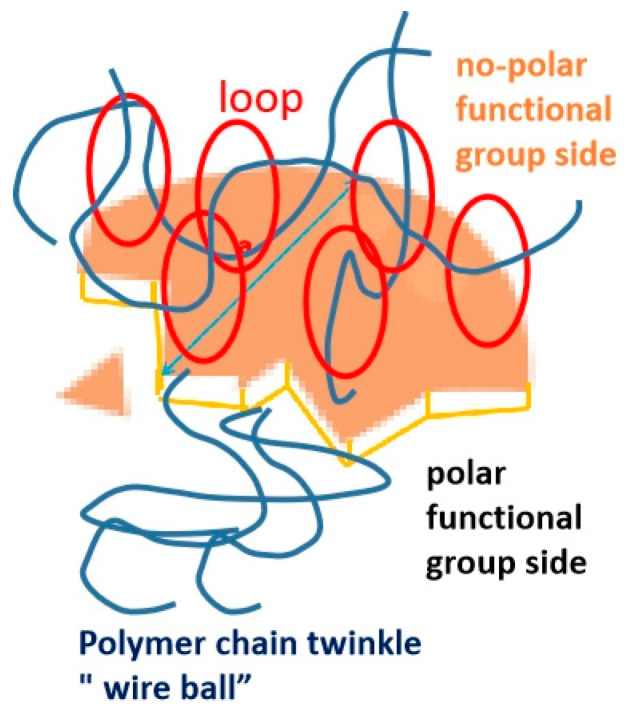
PP molecular chain “loop” on the Janus nanosheet.

After analyzing the viscoelastic experimental results, we have formulated a theory on the correlation between Janus nanosheets and PP molecular chains, as shown in Figure 8. The polar functional group present on the Janus nanosheet strongly attracts the nonpolar PP molecular chains, causing them to adhere to the surface. However, during the melting process, the PP molecular chains repel each other, resulting in an unwinding and stretching motion. On the contrary, the polar functional group on the Janus nanosheet is compatible with the PP molecular chains, leading to the same chain repeatedly coming into contact with the surface, which can even form a loop structure. As more Janus nanosheets are introduced or when they form ”particle clusters” due to agglomeration, the molecular chains become more entangled, thereby significantly enhancing the complex viscosity.

When the amount of Janus B added is extremely small (e.g., 0.05 wt%), clumping becomes less likely. Janus nanosheets are actually smaller than the space where polymer chains entangle. The Janus sheet’s surface attracts the polymer chain, resulting in greater free space at the interface. This change in structure lowers the entanglement density, causing a decrease in complex viscosity.

Even a small amount of Janus S added to PP can cause particle clusters to form. This is due to its larger thickness and tendency to agglomerate. However, increasing the amount of Janus S to ≥0.10 wt% results in a more significant thickening effect compared to Janus B. Additionally, shear thinning occurs earlier. It is crucial to consider these effects when incorporating Janus S into composite systems.

### 3.3. Foam Autoclave Results

Linear viscoelastic properties test results have unequivocally confirmed that both T-PP and NPP exhibit the same complex viscosity. At any addition amount, Janus S to the Janus/PP compound system yields a noticeable increase in viscosity, following a similar pattern as observed in JPP-B3. Nevertheless, it is only Janus B1 that can induce “viscosity reduction” effect in the composite system. N-PP, T-PP, JPP-B1, and JPP-B3 were selected and tested with 99.99 wt% carbon dioxide as the foaming agent at different temperatures and pressures. The resulting foaming bead test results are shown in Table 2.

We randomly picked eight EPP beads and measured their apparent density thrice, taking an average of the results. Similarly, for volume percentage of closed cells, we randomly selected 4–5 mL of EPP beads and tested each sample thrice, calculating the average of the outcomes.

Density. The density of foam material has a significant impact on its properties. Figure 3 displays the densities of various EPP beads and highlights how the use of inorganic particles in autoclave foams can greatly affect density. EPP beads that contain inorganic particles exhibit lower densities than neat N-PP, with density decreasing as process temperature and pressure increase. PP, due to its low viscosity and weak melt strength, has the highest density among N-PP EPP beads. Conversely, PP/nanosheets foams have lower densities as the well-dispersed nanosheets help retain CO_2_ for cell growth, ultimately leading to a decrease in apparent density.

Based on the test results of various samples, it is evident that JPP-B3 EPP beads have the lowest apparent density when processed under the same parameters. It is worth noting that the densities of EPP X9-X16 beads decrease as more Janus nanosheets are added. However, a lower content of Janus nanosheets (0.05 wt%) does not disperse enough to provide sufficient hetero-bubble pore nucleation points in JPP-B1. On the other hand, a higher concentration of Janus nanosheets (0.30 wt%) results in a notable increase in JPP-B3’s complex viscosity, which plays a crucial role in cell growth.

**Figure 9:**
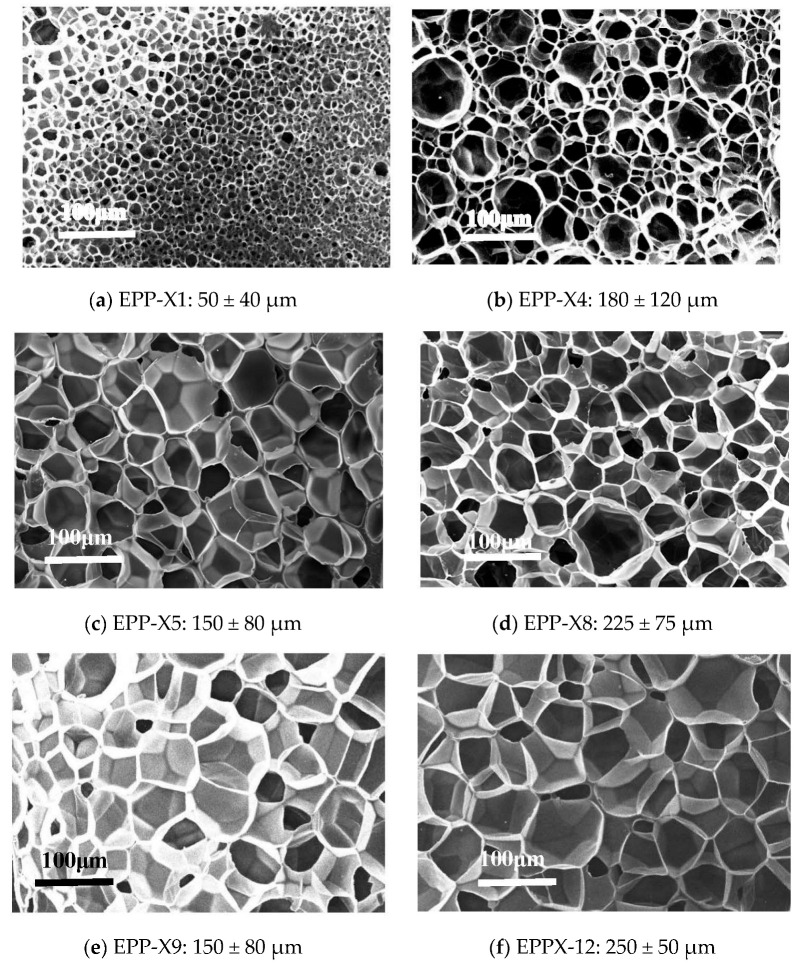

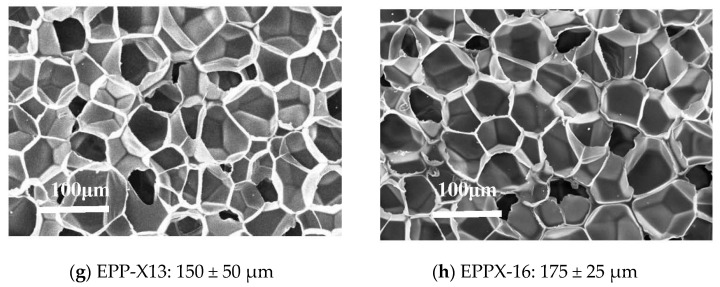
SEM images of EPP beads showing a tendency towards cell structures.

Foam morphology. To analyze the average size and distribution of cells in the foams, we used SEM images. We selected the sample with the smallest cell sizes for comparison, which is displayed in Figure 9. The foams without nanosheets (N-PP) had uneven cell sizes with a broad distribution. As the processing temperature increased, the mean cell sizes and size distribution increased due to the weakening of PP melt strength. All types of nanosheets reduced the mean cell sizes and narrowed the cell size distributions compared to N-PP. However, it is important to note that the cell structure of the foam was significantly affected by the type and amount of additive used.

Janus nanosheets produce smaller and more evenly sized cells than talc when utilizing the same process parameters and 0.30 wt% of additives. This is due to Janus nanosheets being able to incorporate within the molecular chains of PP, increasing its complex viscosity and preventing cell wall coalescence. The quantity of Janus nanosheets utilized impacts the cell sizes, with lower Janus content resulting in smaller cells and a wider distribution present in EPP X13-X16 beads. Comparable cell structures are observed in EPP X9-X12 and EPP X5-X8 beads because of the “non-Einstein effect”, which reduces PP’s complex viscosity. Table 2 and Figure 9 SEM consistently demonstrate trends in volume percentage of closed cells, which is a crucial measure of cell wall integrity in EPP beads.

Our hypothesis is that through rapid pressure relief during EPP bead molding, CO_2_ disperses from the PP melt liquid phase to the gas phase, quickly releasing from the PP microparticles. This process enhances by the Janus nanosheets, allowing for better dispersion of CO_2_ in the EPP beads. This in turn improves melt and interface strength, making the holes more resistant to stretching. The results of the apparent density of EPP beads and obturator rate provide partial confirmation of our speculation.

## 4. Conclusions

The Janus nanosheet has a profound impact on the viscosity of the composite system, far surpassing that of the unmodified inorganic talc. Even a minute addition of 0.10 wt% of Janus nanosheets can significantly increase the complex viscosity, with a more pronounced effect at higher addition amounts. The Janus nanosheet’s thickness plays a crucial role in the viscoelastic modifications of the Janus/PP composite system. The decrease in apparent viscosity only occurs when the Janus nanosheet thickness is below a particular threshold. When introduced in small amounts with minimal thickness, it readily infiltrates the space between the polymer chains, thereby increasing the interface’s free volume and reducing the composite system’s complex viscosity. It is worth mentioning that the Janus sheet’s adhesion effect is not due to a phase state change or a formation of a packing network in the composite system. Instead, the polymer chains on the sheet’s surface tangle, creating a “loop” structure, all because of the different functional groups on both sides of the Janus sheet.

Using Janus nanosheets is highly effective in managing foam structure and decreasing the apparent density of EPP beads, as our research has found. Janus nanosheets outperform talc sheets by resulting in smaller and more uniform cell sizes in the foam, as shown in Figure 8, which displays an SEM image of a cell wall in a foam made from N-PP, T-PP, and JPP-B3. 

The Janus nanosheet is the key to controlling foam morphology and reducing the apparent density of EPP beads. Even with only 0.05 wt% of Janus nanosheets, we were able to improve the volume percentage of closed cells compared to regular PP foam. With 0.30 wt% of Janus nanosheets, we were able to improve the volume percentage of closed cells compared to the same amount of talc sheet modified foam. It is important to note that this improvement is due to the reinforcing effect of the Janus nanosheet, not the foam morphology, density, or complex viscosity increase.

## Figures and Tables

**Table 1:** Overview of the compounded material and pelletizing parameters.

Sample	Additive	Additive Concentration (wt%)	Die Temp (°C)	Feeding (kg /h)	Current (A)	Micropellet Size (mm)
N-PP	—	—	260	13–15	15.6	1 ± 0.1
T-PP	Talc	0.05	260	13–15	15.8	1 ± 0.1
JPP-B1	Janus B	0.05	260	13–15	15.7	1 ± 0.1
JPP-B2	Janus B	0.10	260	13–15	15.7	1 ± 0.1
JPP-B3	Janus B	0.30	260	13–15	15.6	1 ± 0.1
JPP-S1	Janus S	0.05	260	13–15	15.6	1 ± 0.1
JPP-S2	Janus S	0.10	260	13–15	15.7	1 ± 0.1
JPP-S3	Janus S	0.30	260	13–15	15.7	1 ± 0.1

**Table 2:** Summary of characteristic data of different autoclave-foamed PP.

Sample	Material	Temperature (°C)	Pressure (MPa)	Density (g/cm^3^) ^a^	Cell Size (mm) ^b^	Volume Percentage of Closed Cells (%) ^c^
EPP-X1	N-PP	146	3.00	0.075	50 ± 40	89.2
EPP-X2	N-PP	146	4.00	0.062	200 ± 80	87.9
EPP-X3	N-PP	148	3.00	0.061	300 ± 120	84.3
EPP-X4	N-PP	148	4.00	0.051	180 ± 120	80.2
EPP-X5	T-PP	146	3.00	0.054	150 ± 80	94.5
EPP-X6	T-PP	146	4.00	0.045	200 ± 80	93.1
EPP-X7	T-PP	148	3.00	0.044	250 ± 75	91.3
EPP-X8	T-PP	148	4.00	0.037	225 ± 75	89.6
EPP-X9	JPP-B1	146	3.00	0.057	150 ± 80	96.3
EPP-X10	JPP-B1	146	4.00	0.048	200 ± 75	94.3
EPP-X11	JPP-B1	148	3.00	0.048	250 ± 50	92.4
EPP-X12	JPP-B1	148	4.00	0.041	250 ± 50	90.8
EPP-X13	JPP-B3	146	3.00	0.051	150 ± 50	98.7
EPP-X14	JPP-B3	146	4.00	0.040	175 ± 35	97.5
EPP-X15	JPP-B3	148	3.00	0.039	200 ± 35	96.4
EPP-X16	JPP-B3	148	4.00	0.034	175 ± 25	95.1

^a^ As determined by buoyancy method. ^b^ As determined by SEM. ^c^ As determined by method of ASTM D6226-15.

## Data Availability

Data sharing not applicable.
